# Medicare Plans’ Adoption of Special Supplemental Benefits for the Chronically Ill for Enrollees With Social Needs

**DOI:** 10.1001/jamanetworkopen.2020.4690

**Published:** 2020-05-12

**Authors:** David J. Meyers, Emily A. Gadbois, Joan Brazier, Emma Tucher, Kali S. Thomas

**Affiliations:** 1 Department of Health Services, Policy, and Practice, Brown University School of Public Health, Providence, Rhode Island; 2 Center for Gerontology and Healthcare Research, Brown University School of Public Health, Providence, Rhode Island; 3 US Department of Veterans Affairs Medical Center, Providence, Rhode Island

## Abstract

This cross-sectional study assesses the extent to which Medicare plans have offered Special Supplemental Benefits for the Chronically Ill for enrollees with social needs.

## Introduction

In the Medicare Advantage (MA) program, which enrolls 34% of Medicare beneficiaries,^1^ private plans are paid per capita to cover enrollees’ needs. The Creating High-Quality Results and Outcomes Necessary to Improve Chronic (CHRONIC) Care Act of 2018, gave plans new flexibility to offer Special Supplemental Benefits for the Chronically Ill (SSBCI), which address enrollees’ social needs.^2^ Plans have discretion to target these benefits to enrollees with specific chronic conditions.

There is evidence that addressing enrollees’ social needs may be associated with positive outcomes and cost savings.^3^ However, plans have been slow to adopt new benefits.^4^ We analyzed the extent to which plans have offered new SSBCI in 2020.

## Methods

This cross-sectional study used publicly available benefit, plan characteristic, and enrollment files to characterize which plans offered new benefits. Examples of SSBCI newly allowed in 2020 include meal and produce delivery services, nonmedical transportation, pest control, air conditioning, and other benefits to address social needs.^5^ This study did not require institutional review board review because it used publicly available nonhuman participant data. This study followed the Strengthening the Reporting of Observational Studies in Epidemiology (STROBE) reporting guideline.

We compared the characteristics of plans (ie, type, size, star rating, age, nonprofit status) that offered SSBCI in 2020 using 2-sided χ^2^ tests with α = .05. Analyses were performed using Stata, version 15 (StataCorp) in December 2019.

## Results

In 2020, 139 of 3052 plans (4.6%) offered an SSBCI. Pest control (66 plans [2.2%]) was most frequently offered, followed by produce (63 [2.1%]) and meal delivery (55 [1.8%]) (Table 1).

**Table 1.** New Special Supplemental Benefits for Medicare Enrollees With Chronic Illness Offered in 2020^a^

Benefit	With a new SSBCI, No. (%)		
	Contracts (n = 462)	Plans (n = 3052)	Enrollees (n = 20 263 077)
Produce	14 (3.0)	63 (2.1)	416 696 (2.2)
Meals	9 (1.9)	55 (1.8)	208 343 (1.1)
Nonmedical transportation			
Van	10 (2.2)	25 (0.8)	170 698 (0.9)
Taxi	7 (1.5)	16 (0.5)	83 485 (0.4)
Ambulance	6 (1.3)	14 (0.5)	155 658 (0.8)
Ride share	4 (0.9)	13 (0.4)	41 118 (0.2)
Public transit	1 (0.2)	1 (0.0)	41 308 (0.2)
≥2 Transportation options	11 (2.4)	28 (0.9)	238 123 (1.2)
Pest control	10 (2.2)	66 (2.2)	468 592 (2.4)
Air quality control	5 (1.1)	15 (0.5)	202 665 (1.1)
Service dog support	7 (1.5)	37 (1.2)	324 427 (1.7)
Other social need, unspecified	9 (1.9)	22 (0.7)	92 093 (0.5)
Any new SSBCI	33 (7.1)	139 (4.6)	850 698 (4.4)
Any 2 new SSBCIs	19 (4.1)	89 (2.9)	599 789 (3.1)

Abbreviation: SSBCI, Special Supplemental Benefit for the Chronically Ill.

^a^ Data are from the Centers for Medicare & Medicaid Services 2020 quarter 2 plan benefits data and were categorized by us. Enrollee numbers are from 2020 and assigned to corresponding plans in 2020. A given insurance company enters into contracts with the Centers for Medicare & Medicaid Services to offer services, and each contract can contain multiple plans, each with different characteristics. If a plan offered a meal benefit, it denotes that they are offering a meal program beyond what is included under Medicare Part B (eg, short-duration meals after hospitalization). Fully Integrated Medicare-Medicaid, Program of All-Inclusive Care for the Elderly plans, and Medicare Advantage value-based insurance design plans are excluded from this table because state policies influence their benefit design.

Health maintenance organizations (130 plans [6.2%]), plans rated 4 to 4.5 stars (90 [5.4%]), dual (26 [7.5%]) and chronic (27 [24.3%]) special needs plans, and plans created from 2006 through 2013 (57 [5.7%]) were most likely to offer a new SSBCI (all *P* < .001) (Table 2).

**Table 2.** Plans That Offered Any New Special Supplemental Benefit for Medicare Enrollees With Chronic Illness^a^

Variable	With a new SSBCI, No. (%)^b^	
	Plans	Enrollees
Type		
Health maintenance organization	130 (6.2)	821 533 (7.0)
Preferred provider organization	9 (1.1)	29 165 (0.4)
Other^c^	0	0
Enrollment^d^		
Small, <4100	34 (3.6)	9890 (4.0)
Medium, 4100-23 500	59 (5.7)	100 392 (5.6)
Large, >23 500	46 (4.3)	740 416 (4.3)
Star category		
2-2.5	0	0
3-3.5	3 (5.3)	151 390 (5.3)
4-4.5	90 (5.4)	687 141 (6.8)
5	7 (4.0)	11 034 (0.7)
Chronic condition special needs plan	27 (24.3)	43 289 (17.1)
Dual-eligible special needs plan	26 (7.5)	175 689 (8.6)
Contract start time^e^		
Before 2006	73 (4.3)	519 187 (4.1)
2006-2013	57 (5.7)	320 428 (5.8)
2014-2020	9 (2.7)	11 083 (1.2)
Tax status		
For profit	105 (4.6)	636 837 (4.6)
Nonprofit	34 (4.3)	213 861 (3.9)
Census region		
Northeast	25 (3.9)	188 619 (5.1)
Midwest	23 (3.8)	250 992 (7.0)
South	43 (4.0)	257 865 (3.4)
West	47 (7.0)	138 902 (3.3)

Abbreviation: SSBCI, Special Supplemental Benefits for the Chronically Ill.

^a^ Data are from Centers for Medicare & Medicaid Services 2020 quarter 2 plan benefits data. A given insurance company enters into contracts with the Centers for Medicare & Medicaid Services to offer services, and each contract can contain multiple plans, each with different characteristics. Characteristics are reported at the contract level because they do not vary at the plan level. *P* < .01 for all comparisons by Pearson χ^2^ statistic. Fully Integrated Medicare-Medicaid and Program of All Inclusive Care for the Elderly plans are excluded because state policies influence their benefit design.

^b^ Row percentages of the total number of plans and enrollees are shown.

^c^ Other types of plans indicate private fee-for-service, cost, or medical savings account plans.

^d^ Categories of enrollment are based on tertiles of total contract enrollment in 2020.

^e^ The contract start time correspond to the periods before the Medicare Modernization Act (before 2006), between the Medicare Modernization Act and the launch of the Medicare Advantage Quality Improvement Program (2006-2013), and between the Patient Protection and Affordable Care Act and the current year.

## Discussion

Results suggest that MA plans’ adoption of new SSBCI has been limited in 2020, with an estimated 4.6% of plans offering any new benefit. The areas of largest growth are in pest control, produce, and meal programs, which previous work has documented are areas of interest for plans.^6^ Many plans may also have experience offering these benefits through managed Medicaid.

Health maintenance organizations, older plans, and plans with higher ratings more frequently offered new benefits. Older plans may have an established infrastructure that allows for the early adoption of new services. Plans rated 4 stars or higher receive bonuses from the Centers for Medicare & Medicaid Services (CMS) in the form of rebates, which may be used to invest in new services for enrollees.

Regulations detailing what benefits may be covered are generally released in April, and final benefit proposals must be reported to CMS in June. This short period may limit the ability for plans to make decisions in time for the next benefit year.^6^ Limited evidence for the return on investment of these benefits, uncertainties about the extent and parameters of new benefits allowable under the regulation, and the lack of additional funding from CMS for these benefits may also be associated with low adoption.^6^

This study has limitations. Plans use text entry fields to capture new benefits in the benefit files. This analysis may be limited if any of these benefits were misclassified. Given reinterpretation of the uniformity requirement,^5^ we cannot determine how many enrollees had access to a benefit.

The CHRONIC Care Act received substantial attention for encouraging plans to offer benefits to address enrollees’ social needs. However, in the first year, relatively few plans took advantage of this flexibility. It remains to be seen the extent to which these new benefits will become widely available to MA enrollees.
